# Identification of serum biomarker panel to differentiate malignant from benign thyroid nodules using multiplex bead assay

**DOI:** 10.1186/s43046-020-00046-0

**Published:** 2020-09-04

**Authors:** Ragaa Abdelkader Ramadan, Wafaa Ragab, Ramy Samir Assaad, Ahmed Elsayed Shaaban, Amira Ibrahim Fayad

**Affiliations:** 1 Chemical Pathology Department, Medical Research Institute, Alexandria University, Alexandria, Egypt; 2 Department of Experimental and Clinical Surgery, Medical Research Institute, Alexandria University, Alexandria, Egypt; 3 Clinical Pathology Department, Faculty of Medicine, Alexandria University, Alexandria, Egypt

**Keywords:** Cancer thyroid, Thyroid nodule, Epidermal growth factor, Hepatocyte growth factor, Interleukin-8, Monokine induced by interferon gamma, Multiplex bead assay

## Abstract

**Background:**

The challenging target in the workup of thyroid nodule(s) is to exclude or diagnose thyroid cancer efficiently prior to surgical intervention. The present work studied a panel of eight serum biomarkers to differentiate benign from malignant thyroid nodules, aiming at reducing unnecessary thyroidectomy performed for inconclusive preoperative fine needle aspiration cytology.

Serum interleukin-5 (IL-5), interleukin-8 (IL-8), hepatocyte growth factor (HGF), epidermal growth factor (EGF), angiopietin (Ang1), nonokine induced by interferon gamma (MIG), galectin (Gal-3), and vitamin D-binding protein (VDRP) were quantified by multiplex bead assay using Luminex xMAP technology. The study was conducted on 60 subjects of three groups (20 each; healthy controls, benign thyroid nodule, and malignant thyroid nodule).

**Results:**

Significant increase of the following biomarkers in the malignant group compared to the benign group was found; IL-8: 29.7 vs 8.75 pg/ml, *p* < 0.001, EGF: 128.7 vs 6.72 pg/ml, *p* < 0.001, HGF: 173.2 vs 112.2 pg/ml, *p* = 0.012, MIG: 776.7 vs 438 pg/ml, *p* = 0.023, and Ang-1: 95016 vs 33327.5 pg/ml, *p* = 0.014. No significant differences were detected for IL-5, Gal-3, and VDBP. Serum IL-8 and EGF showed the highest diagnostic performance individually with area under the curve (AUC) 0.849 and 0.848, respectively. The combined biomarker panels of IL-8 and EGF and IL-8, EGF, and MIG have reached a sensitivity and specificity of 95% and 65%, respectively, with a negative predictive value of 92.9%.

**Conclusions:**

Serum IL-8 and EGF individually or the combined biomarker panel of IL-8, EGF, and MIG are promising tests that can help to exclude malignancy in thyroid nodule workup.

## Background

The majority of thyroid nodules are benign with approximately 7–15% of the nodules diagnosed as thyroid carcinomas depending on several factors as clinical, environmental, and family history [1, 2]. The National Population-Based Cancer Registry Program in Egypt reported that thyroid cancer represented the 6th commonest cancer among the total Egyptian females accounting for 3.3% of all cancers in 2014 [3].

The clinical significance of thyroid nodules lies in the necessity to exclude thyroid malignancies. The limitations of thyroid fine needle aspiration cytology (FNAC) in distinguishing benign from malignant thyroid lesions lead to unnecessary thyroidectomy in a significant percent of suspicious thyroid nodules that proved by post-operative histopathological examination to be benign [4, 5]. On the other hand, serum marker analysis is a non-invasive technique and precludes many of the immune-cytochemical difficulties.

Oncology research is focusing on the use of a panel of biomarkers rather than a single biomarker diagnostic strategy. Recent laboratory technology like multiplex bead assay has encouraged such studies. Based on literature review, we studied eight biomarkers; interleukin-5 (IL-5), interleukin-8 (IL-8), hepatocyte growth factor (HGF), epidermal growth factor (EGF), angiopietin (Ang1), monokine induced by interferon gamma (MIG), galectin (Gal-3), and vitamin D-binding protein (VDRP). We selected them based on their proven roles in cancer pathogenesis and specific involvement in thyroid oncogenesis. We evaluated their diagnostic ability in discriminating malignant from benign thyroid nodules.

## Methods

### Subject work-up

Sixty Egyptian adults were recruited in the period from August 2016 to June 2017. The included subjects were 40 patients with thyroid nodule(s) as well as 20 healthy volunteers.

All procedures performed in our study were in accordance with the ethical standards of the Ethical Committee of our university and with the 1964 Helsinki Declaration and its later amendments or comparable ethical standards. Informed consent was obtained from all individual participants included in the study.

To all subjects, detailed history was taken, and thyroid clinical examination and ultrasound scan of thyroid and neck were performed. FNAC from the patients with thyroid nodules were preoperatively examined by two independent pathologists and the nodule(s) were classified according to the Bethesda system [1]. Post-operative excisional thyroid biopsy, with or without cervical lymph nodes were doubly examined—grossly and microscopically—by two expert independent pathologists. Based on post-operative TNM classification, patients were classified into those with benign thyroid nodule (20 patients) and malignant thyroid nodule (20 patients).

The following were excluded; diabetes, chronic inflammatory conditions, malignancy other than thyroid, patients who have undergone thyroidectomy, and patients who started therapeutic chemotherapy, thyroid hormone suppression therapy, and radiotherapy.

### Quantitative measurement of the eight selected serum biomarkers using multiplex bead assay (Luminex xMAP® technology)

Peripheral venous blood samples were used to analyze the selected biomarkers by magnetic Luminex assay, Human premixed multi-Analyte kit, Catalog Number LXSAHM, R&D system, USA, using Luminex® 100/200™. Samples were obtained before thyroid surgical intervention. The assay was conducted following manufacturer instructions. The median fluorescence intensity (MFI) of the different calibrator dilutions were used to create a standard curve for each analyte. The concentration of each biomarker was calculated by plotting the corresponding analyte MFI on its standard curve. The assay has a coefficient of variation of 3.7 and 6.8 across the standard curve for intra- and inter-assay precision respectively.

### Statistical analysis

Data were fed to the computer and analyzed using IBM SPSS software package version 20.0 [6]. Significance of the obtained results was judged at the 5% level. Nonparametric analysis was performed using Mann-Whitney, Kruskal-Wallis test, and post hoc (Dunn’s multiple comparisons test, as indicated). Regression analyses were performed for the studied parameters. For diagnostic performance, we applied sensitivity: representing the true positives (TP), specificity: representing the true negatives (TN), positive predictive value (PPV), and negative predictive value (NPV). Receiver operating characteristic (ROC) and the area under the curve (AUC) were used to evaluate the accuracy of the test in the predilection of malignancy. Agreement and diagnostic performance were performed for individual markers and combinations at the cut-offs chosen by the Youden index.

## Results

The studied groups were sex-matched (*p* = 0.75), and also, the benign thyroid nodule (BTN) group and the malignant thyroid nodule (MTN) group were age-matched (46 ± 12.45 vs 44.6 ± 13 years, *p* = 0.745). (Table 1)

**Table 1:** Comparison of sex and age between the studied groups

	Control (***n*** = 20)	BTN (***n*** = 20)	MTN (***n*** = 20)	***H***	***p***
**Sex (male to female)**	16:4	18:2	16:4	1.035	0.750
**Age (years)**					
Mean ± SD	40.9 ± 11.9	45.95 ± 12.45	44.6 ± 12.96	0.883	0.419
Min.–Max	18–59	18–68	16–72		
Median	43	45.5	45		
	*p*_1_ = 0.876, *p*_2_ = 0.893, *p*_3_ = 0.745				

*BTN* benign thyroid nodule, *MTN* malignant thyroid nodule

*H*, *p*: *H* and *p* values for Kruskal-Wallis test, Significance between groups (Sig. bet. gps) was done using post hoc test (Dunn’s multiple comparisons test)

*p*_1_: *p* value for comparing between control and BTN

*p*_2_: *p* value for comparing between control and MTN

*p*_3_: *p* value for comparing between BTN and MTN

*Statistically significant at *p* ≤ 0.05

FNAC reports for the examined thyroid nodules (40 patients) were as follows; 3 patients with Bethesda II which all proved post-operatively to be benign; 6 cases were classified as Bethesda III, of which 1 (16.7%) proved to be malignant; 18 cases were classified as Bethesda IV, of which 6 (33.3%) proved to be malignant; finally, 9 cases were classified as Bethesda V and 4 cases as Bethesda VI, which all proved to be malignant. Interestingly, 17 cases out of the 24 Bethesda III and IV thyroid nodules (71%) proved to be benign by post-operative histopathological examination (Table 2).

**Table 2:** Preoperative fine needle aspiration cytology and postoperative pathological features of patients

		BTN	MTN	Total
**Preoperative FNAC**				
Bethesda (% risk of malignancy)/BTA	**II** (0–3%)/Thy2	3	0	3
	**III** (5–15%)/Thy3a	5	1	6
	**IV** (15–30%)/Thy3f	12	6	18
	**V** (60–75%)/Thy4	0	9	9
	**VI** (97–99%)/Thy5	0	4	4
	Total	**20**	**20**	**40**
**Postoperative pathological diagnosis**				
Final diagnosis	Colloid nodule	3 (15 %)	–	**40**
	Adenomatous goiter	6 (30 %)	–	
	Follicular adenoma	11 (55 %)	–	
	PTC (including FVPTC)	–	17 (85%)	
	FTC	–	3 (15%)	
	Total	**20**	**20**	
LN metastasis	Positive	–	9 (45%)	
	Negative	–	11 (55%)	
	Total		**20**	
Vascular invasion	Positive	–	3 (15%)	
	Negative	–	17 (85%)	
	Total		**20**	

*BTN* benign thyroid nodule, *MTN* malignant thyroid nodule, *FNAC* fine needle aspiration cytology, *LN* lymph node, *PTC* papillary thyroid carcinoma, *FTC* follicular thyroid carcinoma, *FVPTC* follicular variant of papillary thyroid carcinoma

IL-8, EGF, HGF, and MIG showed statistically significant differences between the three studied groups. They were significantly increased in MTN when compared to the BTN and the control group. Ang-1 showed statistically significant differences between the three studied groups. It was significantly increased in MTN when compared to the BTN but showed no significant increase when compared to the control group. IL-5, Gal-3, and VDBP showed no statistical significance. Multivariate analysis showed a significant difference for IL-8, EGF, HGF, and MIG in differentiating BTN from MTN (Table 3). There was no significant difference in the level of the studied biomarkers between malignant cases with or without lymph node metastasis.

**Table 3:** Comparison of serum marker levels between the studied groups

	Control (***n*** = 20)	BTN (***n*** = 20)	MTN (***n*** = 20)	***H***	***p***
**IL-8 (pg/ml)**					
Min.–Max.	3.16–55.0	3.53–16.70	6.72–2059.3	16.364	<0.001^*^
Median	9.01	8.75	29.69		
	*p*_1_ = 0.619, ***p***_**2**_ = 0.001^*^, ***p***_**3**_ < 0.001^*^, ***p***_**4**_ < 0.001^*^				
**EGF (pg/ml)**					
Min.–Max.	1.24–362.7	1.35–498.9	2.14–845.6	13.975	0.001^*^
Median	12.64	6.72	128.7		
	*p*_1_ = 0.356, ***p***_**2**_ = 0.007^*^, ***p***_**3**_ < 0.001^*^, ***p***_**4**_ < 0.01^*^				
**HGF (pg/ml)**					
Min.–Max.	19.36–385.2	32.40–304.0	64.95–434.8	12.931	0.002^*^
Median	98.05	112.20	173.2		
	*p*_1_ = 0.337, ***p***_**2**_ < 0.001^*^, ***p***_**3**_ = 0.012^*^, ***p***_**4**_ = 0.024*				
**MIG (pg/ml)**					
Min.–Max.	99.80–980.0	286.0–1003.0	287.0–6509.0	11.090	0.004^*^
Median	340.4	438.0	776.7		
	*p*_1_ = 0.322, ***p***_**2**_ = 0.001^*^, ***p***_**3**_ = 0.023^*^, ***p***_**4**_ = 0.038*				
**Ang-1 (pg/ml)**					
Min.–Max.	1788–165431	1788.0–196895	11559–298458	6.060	0.049^*^
Median	81248.0	33327.5	95016.0		
	*p*_1_ = 0.248, *p*_2_ = 0.192, ***p***_**3**_ = 0.014^*^				
**IL-5 (pg/ml)**					
Min.–Max.	0.30–1.30	0.07–1.95	0.14–13.51	4.558	0.102
Median	0.68	0.68	1.24		
**Gal-3 (pg/ml)**					
Min.–Max.	4192.0–19988.0	4263.0 16167.0	3759.0–17680.0	0.545	0.761
Median	8146.0	8362.0	8836.0		
**VDBP (ng/ml)**					
Min.–Max.	4854.0–49331.0	16826 73693	19770 69758	0.903	0.637
Median	30334.5	32496.5	29355.5		

*IL* interleukin, *EGF* epidermal growth factor, *HGF* hepatocyte growth factor, *MIG* monokine induced by interferon gamma, *Ang-1* angiopoietin-1, *Gal-3* galectin-3, *VDBP* vitamin D-binding protein, *BTN* benign thyroid nodule, *MTN* malignant thyroid nodule

*H*, *p*: H and *p* values for Kruskal-Wallis test, significance between groups (Sig. bet. gps) was done using post hoc test (Dunn’s multiple comparisons test)

*p*_1_: *p* value for comparing between control and BTN

*p*_2_: *p* value for comparing between control and MTN

*p*_3_: *p* value for comparing between BTN and MTN

*p*_4_: *p* value for comparing between BTN and MTN using multivariate analysis

*: Statistically significant at *p* ≤ 0.05

**Fig. 1:**
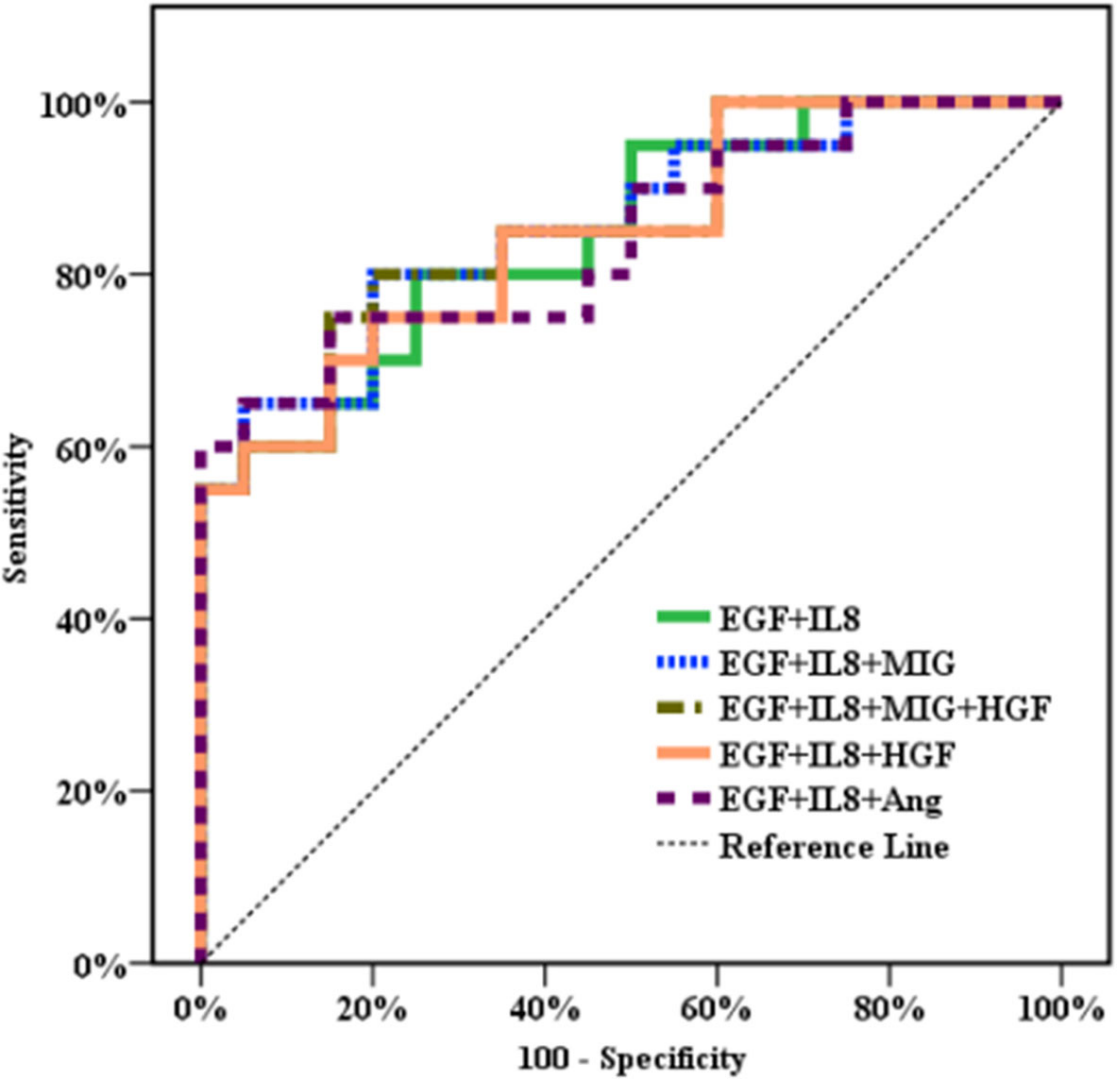
Receiver operating characteristics (ROC) curves for different marker combinations to discriminate malignant from benign nodules. IL interleukin, EGF epidermal growth factor, HGF hepatocyte growth factor, MIG monokine induced by interferon gamma, Ang-1 angiopoietin-1, Gal-3 galectin-3, VDBP vitamin D binding protein

The diagnostic performance of biomarkers to differentiate MTN from BTN was compared against the gold standard tool which is the post-operative histopathological examination. ROC curves were plotted for the five significant biomarkers. Serum IL-8 and EGF showed the highest single marker diagnostic performance with ROC curves AUC 0.849 and 0.848, respectively. The AUC of the different combination panels was compared (IL-8 + EGF + MIG) and (IL-8 + EGF + MIG + HGF) showed the best AUC (0.858) (Fig. 1 and Table 4)

**Table 4:** Analysis of receiver operating characteristic (ROC) curves of serum markers and their combinations

	AUC	95% CI	
		LL	UL
**IL-8**	**0.849**	0.732	0.966
**EGF**	**0.848**	0.716	0.979
**HGF**	**0.728**	0.570	0.885
**MIG**	**0.726**	0.565	0.887
**Ang-1**	**0.725**	0.565	0.885
**IL-8 + EGF**	**0.848**	0.730	0.965
**IL-8 + EGF + MIG**	**0.858**	0.742	0.973
**IL-8 + EGF + HGF**	**0.848**	0.729	0.966
**IL-8 + EGF + Ang-1**	**0.843**	0.719	0.966
**IL-8 + EGF + MIG + HGF**	**0.858**	0.743	0.972

*Ang-1* angiopoietin-1, *AUC* area under the curve, *CI* confidence intervals, *EGF* epidermal growth factor, *HGF* hepatocyte growth factor, *IL-8* interleukin-8, *MIG* monokine induced by interferon gamma, *LL* lower limit, *UL* upper limit

Agreement and diagnostic performance analysis were conducted for the significant biomarkers individually and for the different combinations. Positive and negative status for each biomarker was determined according to its result, above or below the marker cut-off, respectively. In each biomarker combination, “negative” was assigned to the patient who was negative for all the markers of the combination (below the cut-offs) assigned by the Youden index for all the combined markers. We evaluated five different biomarker combinations. The combined biomarker panels of (IL-8 and EGF) and (IL-8, EGF, and MIG) had the most acceptable diagnostic performance. They have reached a sensitivity and specificity of 95% and 65%, respectively, with a negative predictive value of 92.9%. Meanwhile, a sensitivity and NPV of 100% was reached in two combinations (IL-8, EGF, and HGF) and (IL-8, EGF, HGF, and MIG), but this was at the expense of specificity (45%) and PPV (64.5%) (Table 5).

**Table 5:** Agreement and diagnostic performance of the significant serum markers

	BTNN/P	MTN N/P	Sensitivity (%)	Specificity (%)	PPV (%)	NPV (%)	Accuracy
**IL-8 > 11.46**	16/4	5/15	75	80	78.9	76.2	77.5
**EGF > 30.24**	17/3	3/17	85	85	85.0	85.0	85.0
**HGF > 106.2**	10/10	2/18	90	50	64.3	83.3	70.0
**MIG > 623.8**	19/1	9/11	55	95	91.7	67.9	75.0
**Ang-1 > 45797**	12/8	3/17	85	60	68.0	80.0	72.5
**IL8 > 11.46** **EGF > 30.24**	13/7	1/19	95	65	73.08	92.86	80.0
**IL8 > 11.46** **EGF > 30.24** **Ang-1 > 45797**	12/8	1/19	95	60	70.37	92.31	77.5
**IL8 > 11.46** **EGF > 30.24** **MIG > 623.8**	13/7	1/19	95	65	73.08	92.86	80.0
**IL8 > 11.46** **EGF > 30.24** **HGF > 106.2**	9/11	0/20	100	45	64.52	100.0	72.50
**IL-8 > 11.46** **EGF > 30.24** **HGF > 106.2** **MIG > 623.8**	9/11	0/20	100	45	64.52	100.0	72.50

All serum values are expressed in pg/ml

*Ang-1* angiopoietin-1, *AUC* area under the curve, *CI* confidence intervals, *EGF* epidermal growth factor, *HGF* hepatocyte growth factor, *IL-8* interleukin-8, *MIG* monokine induced by interferon gamma, *BTN* benign thyroid nodule, *MTN* malignant thyroid nodule, *N/P* number of “negative/positive” was assigned to patient who was below/above the cut-offs assigned by the Youden index, *NPV* negative predictive value, *PPV* positive predictive value

## Discussion

Identification of biomarkers that can be used in cancer discrimination is a scientific and clinical mandate. In the current study, serum IL-8 and EGF, individually and injunction, expressed the highest diagnostic performance in differentiating MTN from BTN.

Pre-operative FNAC and post-thyroidectomy excision biopsy pathological examination were performed to all cases of thyroid nodules. Seventy-one percent of our patients reported by FNAC as Bethesda III and IV proved later post-operatively to harbor benign lesions. These subjects could have been saved from unneeded thyroidectomy, unless otherwise medically recommended. Such findings are in accordance with the recorded overlap between benign and malignant final diagnoses for the same FNAC categories [1, 5].

In the present study, IL-8 serum levels showed the highest significant increase among MTN compared with BTN and healthy controls. Also, IL-8 revealed a good discriminatory efficacy between both lesions (AUC of 0.849). Such elevated levels of IL-8 were previously demonstrated in thyroid cancer cell lines [7] as well as serum levels of thyroid carcinoma compared to healthy individuals [8, 9]. IL-8 impacts the course of malignancy especially in terms of promoting cellular proliferation [10] through its induced secretion by tumor necrosis factor (TNF)-α produced by cancer cells and tumor infiltrating immune cells [11]. IL-8 showed no significant difference between healthy subjects and those with BTN. Similarly, Krassas et al. reported that IL-8 was not elevated in benign thyroid diseases as Graves’ disease, toxic nodular goiter, and Hashimoto’s thyroiditis [12]. On the other hand, Provatopoulou et al., showed significantly lower values of IL-8 between healthy individuals and thyroid conditions whether benign or malignant [13]. Discrepancy in the results may be attributed to selection criteria and confounding factors in the studied groups. For example, the subjects in Provatopoulou study were mostly Hashimoto’s thyroiditis.

We found a significant increase and effective discriminatory potential in levels of EGF, HGF, MIG, and Ang-1. EGF is a strong activator of follicular thyroid cell proliferation and an enhancer of the migration and invasiveness of thyroid carcinomas [14]. In accordance with our data, Lam et al. 2011 observed increased expression of *EGFR* in thyroid carcinomas when compared with benign thyroid lesions [15]. On the contrary, Eszlinger et al. reported decreased values of EGF in the supernatants of homogenates of hot thyroid nodules compared to their surrounding tissue [16]. However, EGF serum levels in thyroid diseases need further elucidation particularly with the use of different sample types and various methodological techniques. HGF increased serum levels were previously detected in malignant proliferation and benign thyroid lesions shifting to malignancy as in the study by Veselý et al. 2004 [17]. Moreover, a significant increment of Met/HGF receptor expression in thyroid carcinomas was formerly demonstrated [18, 19].

MIG has a proven antitumor activity, achieved by its interaction with CXCR3 receptors on Th1-cells. This drives T and Natural Killer cells to infiltrate tumor and enhance cancer cell death [20, 21]. As to the best of our knowledge, there is no research work on MIG serum levels in thyroid carcinomas matching or contradicting the significant difference found in our study. Ang-1 researches showed inconsistent results varying from increased expression in malignant thyroid lesions [22, 23] to decreased serum levels in thyroid cancers as compared with the control [24] and even no significant difference between thyroid cancer and benign thyroid tumors [25]. The role of Ang-1 in tumor development and angiogenesis requires further insights to establish its exact role.

Our study results showed no significant difference in Gal-3 between the studied groups. This was in accordance with other authors [26], although a significant rise of its level was also reported [27]. Gal-3 has been especially involved in cellular adhesion, and its level correlated with the presence of lymphadenopathies and metastasis [28]. This may explain our observation of Gal-3 levels among papillary thyroid carcinomas (the predominant pathology in malignancy group in our study) where lymphatic and vascular metastases are not common characteristics.

Research work performed by Simonovic et al. 2015 showed that cultured peripheral blood cells of thyroid cancer patients produced significantly higher concentrations of Th2/Th9 cytokines (IL-5, IL-13, and IL-9) than control subjects [29]. However, our study could not confirm these observations for IL-5. We found no significant differences in the studied markers concerning lymph node metastasis. In this context, Ramirez et al. 2000 reported the prognostic role of HGF/c-Met in distinguishing aggressive cancers at higher risk of metastatic dissemination, [30] though no research work is available regarding serum levels of HGF in relation to thyroid tumor invasiveness.

We tried all the possible combinations of the five significant markers in our study in a trial to improve the diagnostic performance. Neither of these combinations has significantly ameliorated the AUC better than the two highest-AUC markers (IL-8 and EGF individually), except for a minimal rise to 0.858 achieved with the three markers combination (IL-8, EGF, and MIG) and the four markers combination (IL-8, EGF, MIG, and HGF).

However, on a deeper view for the aim of an additional diagnostic tool for thyroid nodules, we targeted the negative predictive value (NPV) as the most valuable performance criterion for that purpose. We recommend (IL-8 and EGF) and (IL-8, EGF, and MIG) panels, which achieved the most acceptable diagnostic performance; a sensitivity and specificity of 95% and 65%, respectively, with a negative predictive value of 92.9%. Although a sensitivity and NPV of 100% was reached in (IL-8, EGF, and HGF) and (IL-8, EGF, HGF, and MIG) panels, but this was at the expense of specificity (45%) and PPV (64.5%). Our results showed to be promising, at saving a considerable percent of unnecessary thyroidectomy. But still, the relatively small sample size limited performing correlation studies with the pathological features of the tumor such as tumor stage. Pursuing the studied serum biomarker panels on a larger population of patients with thyroid nodules and involving all types of thyroid malignancy is indicated.

## Conclusion

Serum IL-8 and EGF showed the highest diagnostic performance individually, and the combined biomarker panel of IL-8, EGF, and MIG has reached an agreeable sensitivity and NPV. This introduces it as a possible test to help in excluding malignancy in a thyroid nodule and reducing overdiagnosis of cancer thyroid and subsequent unnecessary thyroidectomy. The multiplex bead assay of combined serum biomarkers is a promising efficient non-invasive diagnostic tool for thyroid cancer and can be of future help in the workup of thyroid nodules.

## Data Availability

The data used or analyzed during the study are available from the corresponding author on reasonable request.

## Abbreviations

Ang-1: Angiopoietin-1
AUC: Area under the ROC curve
DTC: Differentiated thyroid cancer
EGF: Epidermal growth factor
FNAC: Fine needle aspiration cytology
Gal-3: Galectin-3
HGF: Hepatocyte growth factor
IL-5: Interleukin-5
IL-8: Interleukin-8
MIG: Monokine induced by interferon gamma
NPV: Negative predictive value
PPV: Positive predictive value
PTC: papillary thyroid carcinoma
ROC: Receiver operating-characteristic curve
TP: True positive
TN: True negative
VDBP: Vitamin D binding protein
xMAP: Multiplex assay by Luminex Corporation
